# The National Capitol Region’s Emergency Department Syndromic Surveillance System:  Do Chief Complaint and Discharge Diagnosis Yield Different Results?

**DOI:** 10.3201/eid0903.020363

**Published:** 2003-03

**Authors:** Elizabeth M. Begier, Denise Sockwell, Leslie M. Branch, John O. Davies-Cole, LaVerne H. Jones, Leslie Edwards, Julie A. Casani, David Blythe

**Affiliations:** * Johns Hopkins Bloomberg School of Public Health, Baltimore, Maryland, USA; † Virginia Department of Health**,** Richmond, Virginia, USA; ‡ District of Columbia Department of Health, Washington, D.C., USA; § Maryland Department of Health and Mental Hygiene, Baltimore, Maryland, USA

**Keywords:** population surveillance/methods, public health, bioterrorism, emergency services, hospital, dispatch

## Abstract

We compared syndromic categorization of chief complaint and discharge diagnosis for 3,919 emergency department visits to two hospitals in the U.S. National Capitol Region. Agreement between chief complaint and discharge diagnosis was good overall (kappa=0.639), but neurologic and sepsis syndromes had markedly lower agreement than other syndromes (kappa statistics 0.085 and 0.105, respectively).

Syndromic surveillance systems monitor disease trends by grouping cases into syndromes rather than specific diagnoses. U.S. state and local health departments are developing and implementing such systems in hopes of reducing the impact of bioterrorism attacks through earlier detection and action than is possible with traditional diagnosis-based surveillance. The rationale for this approach is that the organisms identified by the Centers for Disease Control and Prevention as high priority categories A and B cause diseases that are rare, often misdiagnosed initially (*1*,*2*), and can have overlapping clinical presentations (*3*). Syndromic surveillance systems may also have secondary benefits, including better disease monitoring after an attack and more rapid detection of naturally occurring outbreaks.

Deciding which data sources to use for syndrome assignment is an important consideration for health departments implementing syndromic surveillance. Several systems use emergency department (ED) chief complaint, discharge diagnosis, or both. We hypothesized that systematic differences between chief complaint and discharge diagnosis might affect syndrome assignment and that characterizing such differences would outline the potential strengths and weaknesses of using each type of data.

The National Capitol Region’s ED syndromic surveillance system, a cooperative effort between Maryland, the District of Columbia, and Virginia, uses chief complaint for syndromic assignment, except for a few hospitals that only provide discharge diagnoses. Differences between these data types are functionally important for this system because not all participating hospitals report every day and the comparison statistic is based on each syndrome’s proportion of all ED visits. Therefore, differences exist in the proportion of each data type contributing to the comparison statistic from day to day. This study was not intended to evaluate the utility of the specific syndrome categorization matrix used by the National Capitol Region system, which is still undergoing refinement.

## The Study

The National Capitol Region’s ED syndromic surveillance system has been operating continuously since September 11, 2001. Each day, the preceding day’s ED logs from up to 25 hospitals are faxed to participating health departments. Depending on the hospital’s routine, these logs provide chief complaint, discharge diagnosis, or both, for ED visits initiated the preceding day. Using a syndrome assignment matrix (Figure) modified from one developed by the Centers for Disease Control and Prevention (T.A. Treadwell, M.K. Glynn, and J. Duchin, unpub. data), all ED visits are coded into one of eight mutually exclusive syndromes: “death,” “sepsis,” “rash,” “respiratory” illness, “gastrointestinal” illness, “unspecified infection,” “neurologic” illness, and “other.” The unspecified infection category was designed to capture infectious illnesses that would be categorized into other system-specific syndromes if additional information were available. The neurologic syndrome was intended to identify meningitis and botulism cases. The “other” category includes visits not consistent with any of the seven specified syndromes. Syndrome assignment is hierarchical, following the order listed above, from death to other, and is based on chief complaint or, if chief complaint is not available, discharge diagnosis.

**Figure:**
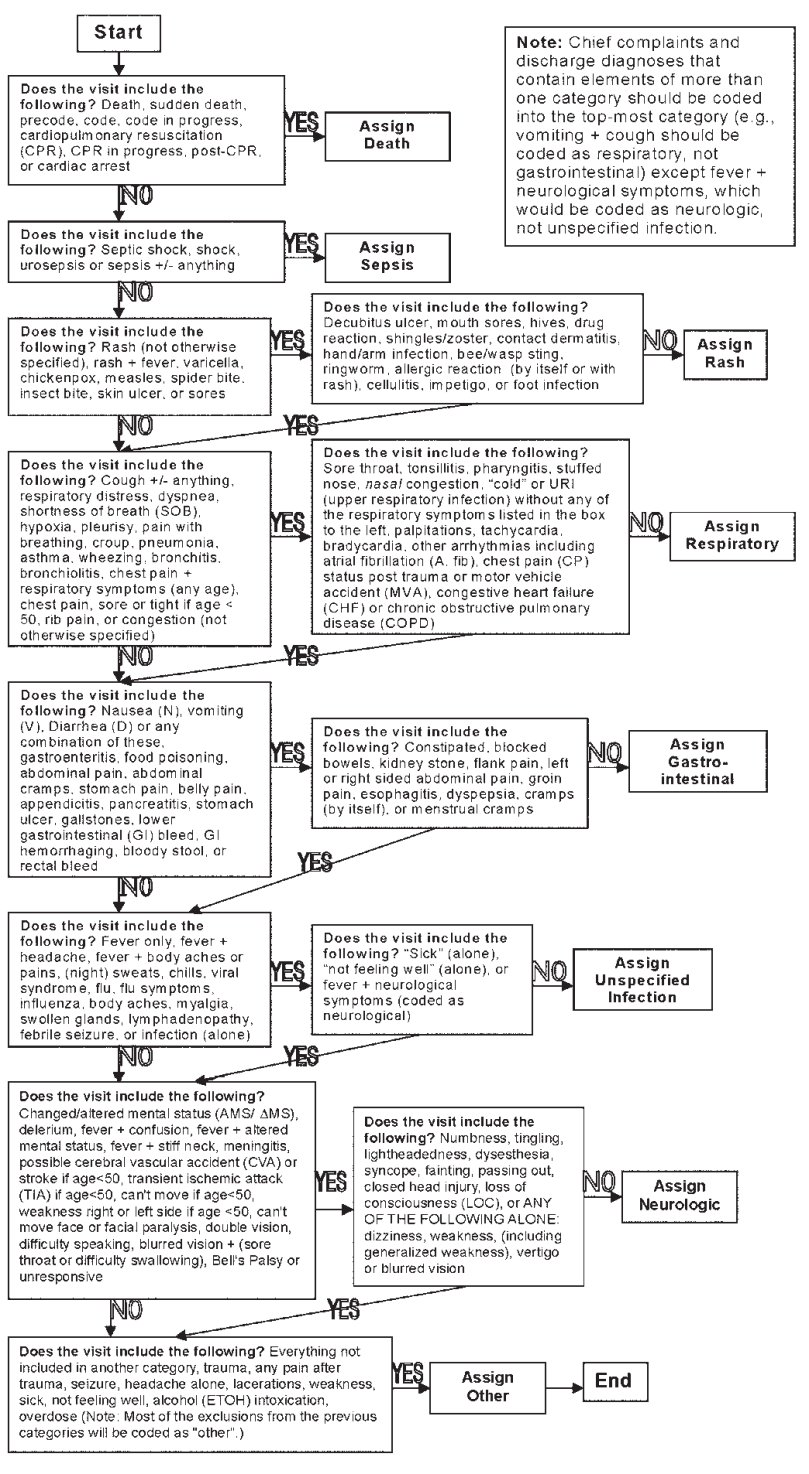
National Capitol Region’s emergency department algorithm for syndrome assignment, United States.

For this study, we used ED logs from two participating hospitals (hospitals 1 and 2) that routinely provide both chief complaint and discharge diagnosis data. Approximately 24,000 and 60,000 ED visits, respectively, occur at these suburban general community hospitals annually. On these logs, both data types are free text because none of the participating EDs assign International Classification of Disease (ICD) codes to visits within 24 hours of ED discharge. In hospital 1, a certified nurse assistant transcribes chief complaints to the log, shortening the chief complaint to a few words from the several sentence version recorded by the triage nurse. The nurse assistant also transcribes the treating clinician’s discharge diagnosis from the medical record to the ED log. At hospital 2, a certified nurse assistant takes and summarizes the patient’s chief complaint in the log. Later, a clinician records the discharge diagnosis using a computerized pick list. These procedures are generally similar in other participating hospitals.

A single trained individual (E.M.B.) reviewed a total of twenty-eight 24-hour ED logs for 14 days in December 2001. Each visit was assigned a syndrome solely on the basis of chief complaint (i.e., the visit’s discharge diagnosis was not viewed) and then rechecked. All logs were then reviewed again to assign each visit a syndrome by using only discharge diagnosis and then rechecked. In all, 4,040 visits were reviewed. One hundred twenty-one visits (3%) were excluded because of missing or illegible chief complaints (n=9), missing or illegible discharge diagnoses (n=100), or both (n=12).

For the 3,919 visits included in the comparison analysis, we calculated overall and syndrome-specific counts, frequencies, and kappa statistics using Stata 7 software (Stata Corp., College Station, TX). All analyses were repeated by hospital. Binary variables for each syndrome for discharge diagnosis and chief complaint were used to calculate kappa by syndrome. Kappa was chosen as the comparison statistic since neither chief complaint nor ED discharge diagnosis accurately provides the patient’s true diagnosis on a consistent basis. Also, kappa corrects for the agreement expected by chance, improving the comparability of the agreement between syndromes of differing prevalence (*4*).

Overall agreement between chief complaint and discharge diagnosis for the 3,919 ED visits compared was good (kappa=0.639, Table 1) (*4*). Respiratory and gastrointestinal syndromes had the highest agreement (kappa statistics 0.684 and 0.677, respectively). The kappa statistic for unspecified infection was in the midrange (0.419). Poor agreement was found for sepsis and neurologic syndromes (kappa statistics 0.105 and 0.085, respectively).

**Table 1:** Relative frequencies of clinical syndromes and kappa statistics for emergency department syndromic-coding results comparing chief complaint vs. discharge diagnosis, National Capitol Region, December 2001^a^

Syndrome	Chief complaint, %	Discharge diagnosis, %	Kappa^a^	Standard error
Death	0.23	0.26	0.6307	0.0160
Sepsis	0.03	0.46	0.1048	0.0071
Rash	1.38	0.79	0.5841	0.0154
Respiratory	13.37	10.61	0.6839	0.0158
Gastrointestinal	13.24	9.26	0.6768	0.0157
Unspecified infection	3.85	2.68	0.4191	0.0157
Neurologic	0.82	0.33	0.0846	0.0145
Other	67.08	75.61	0.6548	0.0156
Overall	–^b^	–^b^	0.6385	0.0104

^a^A total of 3,919 emergency department visits from two regional hospitals were used for all analyses. ^b^Frequencies not applicable to calculation of overall kappa as two categorical variables with eight values, one for each syndrome, were used for this analysis.

Table 2 shows counts of concordant and discordant visits. Sepsis had only one concordant visit, which was the only visit coded as sepsis by chief complaint. Another 17 visits were coded as sepsis by discharge diagnosis only. Seven (41%) were coded as “other” by chief complaint, and the remaining 10 chief complaints (59%) were distributed throughout the syndromes.

**Table 2:** Emergency department visits by syndromic-coding results, by chief complaint and discharge diagnosis, at two U.S. National Capitol Region hospitals, December 2001^a,b^

Syndrome by chief complaint	Syndrome by discharge diagnosis								
	Death	Sepsis	Rash	Resp	GI	UI	Neur	Other	Total
Death	**6**	1	0	0	0	0	0	2	9
Sepsis	0	**1**	0	0	0	0	0	0	1
Rash	0	0	**25**	2	0	2	0	25	54
Respiratory	2	1	0	**339**	6	18	0	158	524
Gastrointestinal	0	1	0	18	**314**	15	1	170	519
Unspecified infection	0	5	0	20	5	**56**	1	64	151
Neurological	0	2	0	1	0	0	**2**	27	32
Other	2	7	6	36	38	14	9	**2,517**	2,629
Total	10	18	31	416	363	105	13	2,963	3,919

^a^Resp, respiratory; GI, gastrointestinal; UI, unspecified infection; Neur, neurologic. ^b^Areas in bold indicate counts of visits with concordant results for both data types.

Neurologic syndrome had 2 concordant visits and 41 discordant visits. Among the 30 discordant visits coded neurologic for chief complaint but not for discharge diagnosis, the most common chief complaint was altered or decreased mental status and level of consciousness (21/30; 70%). Discharge diagnoses for these 30 visits included syncope, sepsis, and other infections; cerebral vascular events or asymmetric weakness in a person >50 years of age; hypoglycemia; and cancer. Eleven discordant visits were coded neurologic for discharge diagnosis only. Nine of these had chief complaints coded “other,” including three patients with Bell’s palsy with chief complaints of facial numbness and three patients with psychiatric chief complaints but discharge diagnoses of change in mental status.

Ninety-five cases were coded as unspecified infection by chief complaint but not by discharge diagnosis. Chief complaints were predominantly “flu” or fever alone or with other nonspecific symptoms. Corresponding discharge diagnoses generally specified the organ system affected and included respiratory infections (bronchiolitis, pneumonia, and bronchitis), gastroenteritis, sepsis, and infections coded as “other” (otitis media, pharyngitis, urinary tract infections, sinusitis, and upper respiratory infection). For the 51 visits coded as unspecified infection for discharge diagnosis but not for chief complaint, the discharge diagnoses were predominately nonspecific terms such as “febrile illness” or “viral illness/syndrome” with syndrome-specific complaints such as cough, vomiting, diarrhea, and rash coded as respiratory, gastrointestinal, or rash.

For three visits, the chief complaints suggested ongoing cardiopulmonary resuscitation and were coded as deaths; however, because all three patients were resuscitated, all had discharge diagnoses other than death. In addition, two visits with chief complaints of respiratory illness and two visits coded as “other” had discharge diagnoses coded as deaths because these four patients subsequently died in the ED.

Hospitals 1 and 2 recorded 971 visits (25%) and 2,948 visits (75%), respectively. Kappa statistics by hospital were similar overall (0.5899 and 0.6504 for hospitals 1 and 2, respectively) and by syndrome, except for rash (0.2822 and 0.6430, respectively) and unspecified infection (0.1786 and 0.4648, respectively).

## Conclusions

Public health officials implementing ED syndromic surveillance systems must decide what data types to use when assigning visits to syndrome categories. Overall we found good agreement between ED chief complaint and discharge diagnosis, but substantial variability existed by syndrome. Sepsis, neurologic, and unspecified infection syndromes were found to have lower agreement than death, rash, respiratory, and gastrointestinal syndromes. These results suggest that several important differences exist between chief complaint and discharge diagnosis.

We found poor agreement between chief complaint and discharge diagnosis for sepsis syndrome. Our matrix terms for sepsis syndrome are sepsis, septic shock, shock, and urosepsis. Sepsis and shock are clinical terms rarely seen as a patient’s chief complaint, even when the ED staff translate patients’ complaints into medical terminology, making this life-threatening clinical entity difficult to track by using chief complaint only.

For neurologic syndrome, which was designed to capture botulism and meningitis cases, we also observed poor agreement between the two data types. Which data source best serves the aims of syndromic surveillance remains unclear, as no botulism or meningitis cases were diagnosed during the study period. Many key components of a meningitis diagnosis are available after a relatively brief ED evaluation, such as classic physical exam findings and spinal fluid analysis results, suggesting that discharge diagnosis may provide a better positive predictive value than chief complaint. Coding initial ED visits of patients with culture-confirmed cases retrospectively, by using chief complaint and discharge diagnosis, would test this hypothesis.

Unspecified infection syndrome is intended to identify nonspecific infectious conditions not captured elsewhere. As expected, we found a low agreement here, since patients with fever alone or other nonspecific chief complaints are often given a specific diagnosis after clinical evaluation. In some situations, organ-specific discharge diagnoses reasonably rule out the possibility of illness caused by category A or B agents. In other situations, these diagnoses may be less informative if they place febrile patients into diagnoses infrequently associated with fever, such as upper respiratory infections, without ruling out serious rare disease.

One limitation of the kappa statistic is its dependence on the prevalence of the condition being detected (*4*). However, the gross differences seen here cannot be accounted for by underlying prevalence. For example, similar prevalences were found for death, sepsis, rash, and neurologic syndromes (range 0.23 to 1.38), but death and rash had substantially higher kappa values than sepsis and neurologic syndromes (0.6307 and 0.5841 vs. 0.1048 and 0.0846, respectively). Another limitation of this analysis is the inability because of sample size to examine interhospital differences in detail. However, kappa statistics were similar for both hospitals overall and differed only by syndrome for unspecified infection and rash. A larger dataset of visits from several hospitals using automated coding would be a better setting for investigating such interhospital variation.

Further work is needed to assess the ability of our syndrome-coding matrix to appropriately classify infections; this matrix continues to undergo refinement. However, our results illustrate important systemic differences between chief complaints and discharge diagnoses. Overall, chief complaint seems to best capture illnesses for which nonspecific symptoms like fever are the most important features. Discharge diagnosis appears better at tracking illnesses that can be identified after brief ED clinical evaluation and testing, such as sepsis and possibly meningitis. Since we are interested in monitoring both types of illness, we recommend coding both data types, if resources allow, or carefully defining system objectives if only one data type can be used. Additionally, linking supplemental clinical information, such as laboratory or radiographic data, to these data sources may substantially improve the predictive value of syndromic surveillance system results overall.
